# MaxGeomHash: An Algorithm for Variable-Size Random Sampling of Distinct Elements

**DOI:** 10.1101/2025.11.11.687920

**Published:** 2025-11-22

**Authors:** Mahmudur Rahman Hera, David Koslicki, Conrado Martínez

**Affiliations:** 1 Center for Advanced Biotechnology & Medicine, Rutgers University, NJ, USA; 2 Department of Computer Science and Engineering, Pennsylvania State University, University Park, PA, USA; 3 Computer Science and Engineering, Biology, and the Huck Institutes of the Life Sciences, Pennsylvania State University, University Park, PA, USA; 4 Department of Computer Science, Universitat Politècnica de Catalunya, Barcelona, Spain

**Keywords:** Random sampling, sketching, *k*-mers, MinHash, FracMinHash, similarity estimation, dimensionality reduction

## Abstract

With the surge in sequencing data generated from an ever-expanding range of biological studies, designing scalable computational techniques has become essential. One effective strategy to enable large-scale computation is to split long DNA or protein sequences into k-mers, and summarize large k-mer sets into compact random samples (a.k.a. *sketches*). These random samples allow for rapid estimation of similarity metrics such as Jaccard or cosine, and thus facilitate scalable computations such as fast similarity search, classification, and clustering. Popular sketching tools in bioinformatics include Mash and sourmash. Mash uses the MinHash algorithm to generate fixed-size sketches; while sourmash employs FracMinHash, which produces sketches whose size scales linearly with the total number of k-mers.

Here, we introduce a novel sketching algorithm, MaxGeomHash, which for a specified integer parameter b≥1, will produce, without prior knowledge of n (the number of k-mers) a random sample of size blg(n/b)+𝒪(b). Notably, this is the first permutation-invariant and parallelizable sketching algorithm to date that can produce sub-linear sketches, to the best of our knowledge. We also introduce a variant, α-MaxGeomHash, that produces random samples of size $\Theta \left(n^{\alpha }\right)$ for a given α∈(0,1). We study the algorithm’s properties, analyze generated sample sizes, verify theoretical results empirically, provide a fast implementation, and investigate similarity estimate quality. With intermediate-sized samples between constant (MinHash) and linear (FracMinHash), MaxGeomHash balances efficiency (smaller samples need less storage and processing) with accuracy (larger samples yield better estimates). On genomic datasets, we demonstrate that MaxGeomHash sketches can be used to compute a similarity tree (proxy for a phylogenetic tree) more accurately than MinHash, and more efficiently than FracMinHash.

Our C++ implementation is available at: github.com/mahmudhera/kmer-sketch. Code to reproduce the analyses and experiments is at: github.com/KoslickiLab/MaxGeomHash.

## 1 Introduction

The exponential growth of genomic and metagenomic sequencing data has necessitated the development of scalable computational methods for efficiently processing and comparing biological sequences. Central to many methods is the concept of k-mers (substrings of length k), which serve as fundamental units for sequence comparison and analysis. However, the vast number of distinct k-mers in modern datasets makes exact comparisons computationally prohibitive, driving the need for approximation techniques that trade accuracy for improvements in speed and memory.

Sketching methods have emerged as a powerful solution, creating compact “fingerprints” of sequence data that preserve essential similarity information while reducing computational requirements. MinHash [1, 22] has become ubiquitous in genomic applications, enabling rapid all-versus-all comparisons through fixed-size sketches. However, as we and others have shown [16, 17], traditional MinHash exhibits significant limitations when comparing sets of very different sizes, a common scenario in metagenomic analyses where bacterial genomes are compared against complex environmental metagenomic samples.

FracMinHash addresses these limitations by allowing sketch sizes to scale with the data [11, 13]. Rather than maintaining a fixed number of hash values, FracMinHash retains a fraction s∈[0,1] of all k-mers. This linear scaling of sketch size enables accurate containment estimation between sets of arbitrary sizes.

While FracMinHash provides excellent accuracy, the price it pays is maintaining very large samples (linear in the number of distinct elements). In modern bioinformatics, genomic repositories and metagenomic studies often contain billions to trillions of sequences, resulting in very large sketch sizes. This motivates the need for new sampling algorithms that can achieve a middle ground: maintaining the accuracy benefits of letting samples grow, while keeping sample sizes sub-linear.

In this work, we propose a novel algorithm for random sampling, which we call MaxGeomHash (MGH for short), formulated as subsampling from a large finite dataset or data stream. Given a user-defined parameter b, our algorithm produces random samples of (expected) size blg(n/b)+𝒪(b) from a data stream containing n distinct elements (where n is possibly unknown), providing a compelling balance between sample size and accuracy. We also present a variant, α-MaxGeomHash (α-MGH for short) that renders samples of expected size $\Theta \left(n^{\alpha }\right),\;\alpha \in (0,1)$, where α is a user-specified parameter.

Importantly, MaxGeomHash and α-MaxGeomHash are the first dependable, order-independent, parallelizable, sub-linear sampling methods to date. Unlike existing sub-linear methods such as Affirmative Sampling [18], which is sensitive to the order of data stream processing and cannot be reliably parallelized, our approach produces identical samples regardless of data partitioning or thread execution order.

We theoretically characterize the expectation and variance of MGH and α-MGH sample sizes and quantify their computational cost rigorously. Our analyses also prove that MGH and α-MGH samples allow for unbiased or asymptotically unbiased similarity estimation, including Jaccard, cosine, and other metrics, by leveraging results from [19, 25].

Empirical evaluations on simulated and real genomic data confirm our theoretical predictions and demonstrate practical utility, including efficient pairwise similarity computation. We show that MGH and α-MGH allow for a balance between MinHash and FracMinHash, with provisions for a better accuracy-vs-efficiency tradeoff. Lastly, we provide an efficient C++ implementation for computing and comparing MGH/α-MGH samples directly from FASTA/FASTQ files, making our methods accessible to the bioinformatics community.

## 2 Background: Random Sampling from Data Streams

Random sampling has a long history in algorithms and data systems, from early treatments and classic textbook expositions [7, 14] to scalable streaming variants such as Reservoir Sampling [31] and its weighted forms [5]. In large-scale bioinformatics, sketching-based sampling is indispensable for scalable, diversity-aware analysis. Prominent families include MinHash and bottom-k [1, 3], FracMinHash [9, 12], and Affirmative Sampling [18]; in biological contexts, FracMinHash-style sketches are particularly effective and can be used to estimate a variety of biologically relevant quantities [8, 9].

### Model and objective.

We observe a long dataset or data stream $𝒵=z_{1},z_{2},\ldots ,z_{N}$ of items drawn from a large *universe* 𝒟, with $z_{i}\in 𝒟$. Let the multiset of distinct elements underlying 𝒵 be $ℳ=\left\{x_{1}^{f_{1}},\ldots ,x_{n}^{f_{n}}\right\}$, with $f_{i}$ the frequency of $x_{i}$ in 𝒵, and let the set of distinct elements be $𝒳=\left\{x_{1},\ldots ,x_{n}\right\}$. The (unknown) number n=|𝒳| is the *cardinality* of 𝒵. Our goal is to obtain a *random sample* 𝒮 of distinct elements from 𝒳 such that, for S=|𝒮|, every S-subset (a subset containing S elements) of 𝒳 is equally likely to be drawn. We focus on methods that operate in one pass over 𝒵 without prior knowledge of n.

### A unifying hashed-sampling view.

Most random sampling algorithms work by sequentially processing the dataset or data stream 𝒵, one element at a time, and keeping a current sample 𝒮 (in all the cases that we study here 𝒮 is a random sample from the subset of elements processed so far). If the current item z is present in 𝒮, we can update its frequency count and move on to the next. If z is not present, we compute its hash value h=h(z) and use h to decide whether z is added to the sample or discarded. Moreover, and depending on our decision w.r.t. z, we might decide to make additional changes in 𝒮, for example, removing some elements from 𝒮. Examples of algorithms that fall into the general scheme outlined above include bottom-k and MinHash [1]^5^ FracMinHash [8, 9, 12], ModHash [1], and Affirmative Sampling [18].

In FracMinHash, an item z is sampled (kept in the sample 𝒮) iff h(z)≤s for a given real value s∈(0,1), where h(.) produces real hash values in the range [0,1]. In ModHash, an item z is sampled iff h(z) mod M=0, where h(.) produces integer hash values in some range [0,H] for some large H. In bottom-k, an item z is sampled iff it is among the k smallest hash values seen so far. If sampling z results in |𝒮|=k+1, then the item with the largest hash value in 𝒮 is removed. MinHash applies k independent hash functions, and an item z is sampled if $h_{i}(z)$ is the minimum hash value among the $h_{i}$ values observed so far, for some i. Affirmative Sampling —the standard variant— samples an item z if not present in the sample and if h(z) is larger than ${min}_{x\in 𝒮}\;h(x)$; moreover, it evicts the item $z^{*}$ with the smallest hash value in 𝒮 if h(z) is not among the b-largest hash values in 𝒮. Initially, the first b items are always included in 𝒮; and at any given moment, the sample 𝒮 will contain |𝒮| distinct elements with largest hash values seen so far.

In FracMinHash and ModHash, the expected size of the sample 𝒮 is linear in n. For FracMinHash, it will follow a binomial distribution of parameters n and s. Likewise, ModHash produces random samples with their size following a binomial distribution of parameters n and 1/M. Last but not least, in Affirmative Sampling, the expected size of 𝒮 is ∼bln(n/b), for a fixed parameter b, in the standard variant; in α-Affirmative Sampling, 0<α<1, the expected size of the sample is $\Theta \left(n^{\alpha }\right)$.

MinHash has the advantage of producing samples of fixed size and the samples/sketches are small and easy to obtain. However, inference based on those sketches is less accurate, especially when they are used to estimate containment and similarity indices between two sets. Conversely, FracMinHash and ModHash give excellent accuracy, but the price they pay is to keep very large samples (linear in the number of distinct elements). Midway, we have Affirmative Sampling, which guarantees very good accuracy and (asymptotically) unbiased estimation, while producing significantly smaller samples (of size Θ(logn) or $\Theta (\sqrt{n})$, say).

All these algorithms return random samples of distinct elements, which is easily provable under pragmatic assumptions on the randomness and independence of the hash functions. Indeed, if all decisions (to sample/to evict/to discard) are solely based upon the hash values of the items, then the samples will be random.

### Dependability.

A sampling algorithm is *dependable* [18] if it allows for exact frequency counts: membership of $x_{j}$ in 𝒮 is fixed at its first occurrence, and items once removed are never reinserted. Reservoir sampling [31] is not dependable, whereas all the other algorithms mentioned are.

### Parallel composition.

Massive datasets must be processed in parallel by partitioning 𝒵 into p disjoint data subsets or substreams ${𝒵}_{1},\ldots ,{𝒵}_{p}$. For order-independent sketches there is a standard merge operation: compute a local sample ${𝒮}_{i}$ from each ${𝒵}_{i}$ and then aggregate $𝒮=\phi \left(\underset{1\leq i\leq p}{⋃}\;\;{𝒮}_{i}\right)$, where ϕ is the algorithm-specific pruning rule (e.g., keep the k smallest hash values, or keep all items with h(x)≤s). This equivalence works both ways: running the algorithm on the entire stream 𝒵 or merging local sketches yields the same distribution. MinHash, bottom-k, FracMinHash, and ModHash satisfy this property; Reservoir Sampling (with repetitions) and Affirmative Sampling (both variants) do not, and are not mergeable in this sense.

### Expected cost of random sampling.

The *cost* of running a sampling algorithm refers to the number of operations required to scan the entire data stream and produce the random sample. We give the expected cost M(n,N) of the random sampling algorithms in the form Θ(N+u(n)·t(S)), where N is the size of the dataset (or the length of the data stream), n the number of distinct elements (n≤N, in many applications n≪N), u(n) is the expected number of times there is an update of the sample (and of auxiliary data structures, if any), and t(S) is the time to update a sample of size S. This cost is based on the assumption that we can test whether a given item z (or hash value h=h(z)) is already present in the sample or not in expected constant time. The expected number of updates u(n) will be at least S, but it is often the case that u(n)≫S, for example, in bottom-k we have S=k, but u(n)=kln(n/k)+𝒪(k) [18]; using a min-heap to keep and efficiently update the sample we have t(S)=𝒪(logk). This gives a total expected cost for bottom-k of 𝒪(N+klogklog(n/k)). Alternately, the cost of producing a FracMinHash sketch is 𝒪(N+ns), as u(n)=S=n·s and t(S)=Θ(1). The expected cost of the new algorithms, MGH and α-MGH, will be investigated in Sections 3 and 4, respectively, where we describe and study their main properties.

### At-a-glance comparison.

We summarize the main properties that guide the choice of a sampler in practice. Dependability is necessary if exact frequency counts are required; mergeability enables distributed processing; the parameter controls the memory footprint; and the last column reports the expected sample size (asterisks indicate quantities that are either fixed or bounded by a constant).

**Table:** 

Algorithm	Dependable?	Mergeable?	Parameter	Expected |𝒮|

MinHash [1]	yes	yes	k∈N	k(*)
ModHash [1]	yes	yes	m∈N	n/m
Reservoir Sampling^6^[31]	no	no	k∈N	≤k(*)
FracMinHash [12]	yes	yes	s∈(0,1)	n⋅s
Affirmative Sampling [18]	yes	no	b∈N	bln(n/b)+𝒪(b)
α-AS [18]	yes	no	α∈(0,1)	$\Theta \left(n^{\alpha }\right)$

MGH (this paper)	yes	yes	b∈N	$b\;{log}_{2}(n/b)+𝒪(b)$
α-MGH (this paper)	yes	yes	α∈(0,1)	$\frac{2^{1/(1-\alpha )}-1}{2^{\alpha /(1-\alpha )}-1}\cdot n^{\alpha }+𝒪(log\;n)$

In summary, our algorithm fits neatly between the fixed-length samples of MinHash and the linearly-sized samples of FracMinHash, while retaining mergeability (which is absent from Affirmative Sampling).

## 3 MaxGeomHash

Here, we describe our algorithm, MaxGeomHash (MGH for short) which is dependable and parallelizable in the sense explained in the previous section. Like Affirmative Sampling, it has the nice property of returning samples of variable size, growing with n. In particular, the standard variant of the algorithm, which we examine now, returns samples of expected size blg(n/b)+𝒪(b)—here, and in the sequel we use $lg\equiv {log}_{2}$.

Given a bit string w≠000⋯0, let zpl(w) denote the length of the longest prefix of 0s in w (if w=0000⋯ then zpl(w)=|w|, by convention). In other words, if zpl(w)=j then the first j bits of w are 0 (bits 0 to j-1), and bit j is 1. We will also use tail (w,j) to denote the bit string that results from removing the first j+1 bits of w (that is, the longest prefix of zeros and the leftmost 1).

For each data stream item z, we compute its hash value h:=h(z), and determine the position i=1+zpl(h) of the leftmost 1 in the binary representation of h. Here we assume that the hash function h(·) maps an item z to an integer h(z) in the range [0,H-1] for some large H (in practice, we used 64-bit hash functions, and $H=2^{64}$ in this case). We also set $h^{\prime }$ as the suffix after the leftmost 1. We use i to index z into a bucket ${𝒮}_{i}$. If ${𝒮}_{i}$ already contains z or $h^{\prime }$ is not among the b largest hash values in ${𝒮}_{i}$ then we will discard z. If ${𝒮}_{i}$ doesn’t yet contain b elements or $h^{\prime }$ is among the b largest hashes in ${𝒮}_{i}$ then we add z to ${𝒮}_{i}$; in the later case, the element $z^{*}$ with the smallest hash value $h^{\prime }$ is evicted, so that ${𝒮}_{i}$ will contain the ≤b elements in the data stream such that their leftmost 1 is at position i of h(z) and have largest $h^{\prime }\;=\;tail(h(z),i)$. A frequency counter is kept for all z∈S. The steps of MaxGeomHash are detailed in Algorithm 1. A visual overview of MaxGeomHash is given in Figure 5.

Let $x_{1},\ldots ,x_{n}$ denote the distinct elements in 𝒵. Let $\rho_{i}=1+zpl\left(hash\left(x_{i}\right)\right)$. The $\rho_{i}$ are i.i.d. geometric random variables with parameter 1/2. Therefore, the probability that an item z has to be considered for bucket ${𝒮}_{i}$ is $1/2^{i}$. Using this, we can compute the expected sample size and variance.

### Theorem 1.

*MaxGeomHash with parameter* b≥1 *will produce a random sample* 𝒮 *of size* $S=|𝒮|=\sum_{1\leq i\leq R}\;\left|{𝒮}_{i}\right|$ *such that*

$$E[S]=E\left[\sum_{i=1}^{R}\;\;\left|{𝒮}_{i}\right|\right]=b\;lg(n/b)+b+\epsilon_{n,b}$$

*where* $R=max\left\{\rho_{j}|1\leq j\leq n\right\}$ *and* $\epsilon_{n,b}\leq E_{b}+o(1)$ *with* $E_{b}=\sum_{m=0}^{b-1}\;(b-m)e^{-b}\frac{b^{m}}{m!}$. *Moreover*, V[S]=Θ(1).

**Algorithm 1:** MaxGeomHash algorithm

procedure MaxGeomHash(Z : data stream; S : sample)
R:= 0
for all z in Z do
h: = hash(z) // h = 0...0 1 h’, h’ = tail(h, 1+zpl(h))
i:= zpl(h)+1; // position of leftmost 1, 1 <= i <= |h|+1
h’:= tail(h, i);
if bucket[i] does not exist then
create bucket[i] and add z to bucket[i] with freq = 1 and hash h’
if i > R then R:= i
elseif z in bucket[i] then
freq(z):= freq(z)+1
elseif |bucket[i]| < b or h’ is among the b largest hashes
add z to bucket[i] with freq = 1 and hash h’
if |bucket[i]| = b+1 then
evict element with the smallest hash from bucket[i]
endif
endif
enfor
return S = {bucket[i] | 1 <= i <= R}
endprocedure

#### Proof (Sketch of the proof).

We give here only an intuition of the proof for E[S]. A fully detailed and rigorous proof can be found in Appendix B.

Each bucket ${𝒮}_{i}$ receives (but only keeps up to b) a certain number $X_{i}∼Binomial\left(n,1/2^{i}\right)$ of items from the data stream 𝒵, that is, $S_{i}=\left|{𝒮}_{i}\right|∼min\left(b,Binomial\left(n,1/2^{i}\right)\right)$ and the indices of buckets run from 1 to R, with $R=max\left\{\rho_{1},\ldots ,\rho_{n}\right\}$. We know E[R]=lgn+𝒪(1) and V[R]=Θ(1) [29]. For the size of the final sample 𝒮 we have $S=|𝒮|=\sum_{i=1}^{R}\;S_{i}$, and because of linearity of expectations and the fast decay of the terms with i≥lgn, it is not difficult to prove that

$$E[S]=E\left[\sum_{i=1}^{R}\;\;E\left[S_{i}\right]\right]=\sum_{i\geq 1}\;E\left[S_{i}\right]P\{R\geq i\}=\sum_{i=1}^{E[R]}\;E\left[S_{i}\right]+𝒪(1).$$

For i≲lg(n/b) we will have $S_{i}=b$ with high probability; likewise, for $i≳i^{*}=lg(n/b)$, $S_{i}=min\left\{X_{i},b\right\}=X_{i}$ with high probability, hence $E\left[S_{i}\right]=n/2^{i}$ and so $\sum_{i\geq lg(n/b)+1}\;E\left[S_{i}\right]=b+o(1)$. The constant $E_{b}$ stems from a constant number of terms in the sum around the critical value $i^{*}$. Summing up, we get the asymptotic estimate given in the statement of the theorem, after carefully considering the different errors in the approximation and bounding them. □

The constant $E_{b}$ in the error bound given in Theorem 1 depends only on b, and its value is very small, so we can easily dismiss the error from a practical standpoint. For example, $E_{2}=3e^{-2}\approx 0.406$ and $E_{5}\approx 0.615$; for moderately large b we have $E_{b}\ll b$, and the error is much smaller than the main term blg(n/b)+b.

The expected cost $M_{b}(n,N)$ of MGH when processing a data stream of N items such that n of them are distinct is dominated by the time needed to scan the data stream 𝒪(N), plus the time to update the buckets i that contain b elements. We expect $N/2^{i}$ items to be directed to bucket i, but most will be discarded with cost Θ(1), as they are repetitions or because their hash $h^{\prime }$ is not among the b larger in ${𝒮}_{i}$. Like in a bottom-k approach, we expect $∼b\;ln\left(n/b2^{i}\right)$ updates in bucket ${𝒮}_{i}$, and the cost of each of these updates is 𝒪(logb). Summing over all possible i, we get that the expected cost is

$$M_{b}(n,N)\in 𝒪\left(N+b\;log\;b\;{log}^{2}(n/b)\right).$$

## 4 α-MaxGeomHash

We now discuss a variant of MGH, called α-MGH, that renders samples of expected size $\Theta \left(n^{\alpha }\right)$ for a fixed parameter α∈(0,1) of our choice. The algorithm is exactly as Algorithm 1, but instead of keeping at most b distinct elements on each ${𝒮}_{i}$, we will keep up to $\left\lceil 2^{\beta i}\right\rceil$ in bucket i, with $\beta :=\frac{\alpha }{1-\alpha }$. Additional fine tuning can be introduced by setting the maximum capacity of each bucket to $\gamma \cdot 2^{\beta i}$, for a given constant γ>0, but for the rest of the section we will stick to γ=1.

The intuition is that bucket i, for small i, namely, i<i*=(1-α)lgn, will receive a lot of elements ($∼n/2^{i}$) but only will keep very few ($2^{\beta i}\ll n/2^{i}$), whereas for large i,i>i*, there are very few items going to bucket i but all of them will be kept $\left(n/2^{i}\ll 2^{\beta i}\right)$. For i*=(1-α)lgn, and since α=β/(1+β) and $2^{i*}=n^{1-\alpha }$, it follows that ${𝒮}_{i*}$ will contain at most $2^{\beta \cdot i*}=n^{\beta /(1+\beta )}=n^{\alpha }$ elements, but on the other hand we expect that $n/n^{1-\alpha }=n^{\alpha }$ distinct elements are directed to bucket i* and kept since $n/2^{i*}=2^{\beta i*}$. Hence $E\left[S_{i*}\right]∼n^{\alpha }$. The accumulated contribution of the other buckets with values of i≠i* can be shown to be $𝒪\left(n^{\alpha }\right)$. Actually, a more delicate analysis yields the following theorem.

### Theorem 2.

*The variant* α-*MGH of MaxGeomHash with parameter* α∈(0,1) *will produce a random sample* 𝒮 *of size* S *such that*

$$E[S]=\left(\frac{2^{1/(1-\alpha )}\;-\;1}{2^{\alpha /(1-\alpha )}\;-\;1}\right)\cdot n^{\alpha }+𝒪(log\;n)\approx \left(\frac{1\;-\;\alpha }{\alpha \;ln\;2}+\frac{3}{2}+\frac{\alpha \;ln\;2}{12(1\;-\;\alpha )}\right)\cdot n^{\alpha }.$$

*Moreover* $V[S]=\Theta \left(n^{\alpha }\right)$.

The proof can be found in Appendix C and runs along similar lines as that of Theorem 1.

We give the expected cost of α-MGH, $M_{\alpha }(n,N)$ in the form of, as we have discussed for the other algorithms, 𝒪(N+u(n)t(S)). The expected total number of updates u(n) is $𝒪\left(n^{\alpha }\right)$ and we can bound the cost of updating any bucket by $𝒪\left(log\left(n^{\alpha }\right)\right)=𝒪(log\;n)$, one therefore has the expected cost of

$$M_{\alpha }(n,N)\in 𝒪\left(N+n^{\alpha }log\;n\right).$$

## 5 Similarity estimation with MGH and α-MGH

Given two sets A and B and random samples $𝒮={\left\{{𝒮}_{i}\right\}}_{1\leq i\leq R_{A}}$ and $𝒯={\left\{{𝒯}_{i}\right\}}_{1\leq i\leq R_{B}}$, we will use them to estimate Jaccard(A,B)=|A∩B|/|A∪B|. We use $A_{i}$ and $B_{i}$ to denote the subsets of A and B of elements x with zpl(x)+1=i. If we use the same hash function to sample from A and from B, then if an item x∈A is considered for bucket ${𝒮}_{i}$ then it will be also considered for bucket ${𝒯}_{i}$ if x also appears in B, and vice versa. By definition, we have $A_{i}\cap A_{j}=\emptyset$ whenever i≠j, and $\underset{i\geq 1}{⋃}\;A_{i}=A$; likewise, ${\left\{B_{i}\right\}}_{i\geq 1}$ gives a partition of B. One important result 19 establishes that if A∩B=∅ then 𝒮+𝒯 is a random sample of A+B (this also holds for ${𝒮}_{i}+{𝒯}_{i}$). But ${𝒮}_{i}\cup {𝒯}_{i}$ is not a random sample of $A_{i}\cup B_{i}$, nor 𝒮∪𝒯 a random sample of A∪B. Therefore, we need to apply a “filtering” or “refining” step. In particular, we will keep only the b elements with larger hash values (à la bottom-k). So for each i, we will compute ${𝒮}_{i}\cup {𝒯}_{i}$ but keep the b elements with larger hash values $h^{\prime }$ (or only $\left|{𝒮}_{i}\cup {𝒯}_{i}\right|$ elements if that cardinality is smaller than b). Let us call this new sample ${𝒰}_{i}$. This is indeed a random sample of $A_{i}\cup B_{i}$. Moreover, we will compute ${𝒱}_{i}={𝒮}_{i}\cap {𝒯}_{i}$. Notice that $\left|{𝒱}_{i}\right|\leq b$, since both ${𝒮}_{i}$ and ${𝒯}_{i}$ contain at most b elements each.

All ${𝒰}_{i}$ are mutually disjoint $\left({𝒰}_{i}\cap {𝒰}_{j}=\emptyset \right.$ if $\left.i\neq j\right)$ which entails that $𝒰=\underset{1\leq i\leq R_{A\cup B}}{⋃}\;{𝒰}_{i}$ is a random sample of A∪B. Likewise $𝒱=\underset{i}{⋃}\;{𝒱}_{i}=A\cap B\cap 𝒰$, hence (see 19 for a detailed proof) |𝒱|/|𝒰| is an unbiased estimator of Jaccard(A,B). In fact, we can use 𝒰 and 𝒱 to estimate many other set similarity and set containment measures [19].

The arguments also work *verbatim* if instead of buckets with maximum capacity equal to b, their capacity is given by $f_{i}$, e.g., $f_{i}=\left\lceil 2^{\beta i}\right\rceil$, as in α-MGH. When computing ${𝒰}_{i}$ we would just need to do the unions ${𝒮}_{i}\cup {𝒯}_{i}$, then retain the elements with the $f_{i}$ larger $h^{\prime }$ values (or less if there are not $f_{i}$ elements in ${𝒮}_{i}\cup {𝒯}_{i}$).

In order to merge two samples 𝒮 and 𝒯 we should proceed as described above, taking advantage that the two samples are already classified into buckets, and computing the unions/intersections of the buckets ${𝒮}_{i}$ and ${𝒯}_{i}$ while only keeping the b (or $f_{i}=\left\lceil 2^{\beta i}\right\rceil$) elements with larger $h^{\prime }$ hash values, doing it in one single pass. But from the point of view of the result, the process is equivalent to applying MGH (or α-MGH) to 𝒮∪𝒯, or 𝒮∩𝒯, to get the union or the intersection of the two samples; indeed, we have the following important property^7^:

$$\begin{array}{rr} & MGH(A\cup B)=MGH(MGH(A)\cup MGH(B)),\\ & MGH(A\cap B)=MGH(MGH(A)\cap MGH(B)). \end{array}$$

Notice that MinHash and FracMinHash satisfy the analogous equalities.

From there, the results of [9, 19, 25] apply, showing that we can use MGH or α-MGH samples to get (asymptotically) unbiased estimates of the Jaccard similarity of A and B, of their cosine similarity, of the containment index, and many other similarity measures, like Kulczinsky 1, Kulczinsky 2, or Sørensen-Dice.

For some similarity measures like Jaccard, containment index, or Kulczinsky 2, the estimates are unbiased; for others, like cosine or Kulczinsky 1, the bias is 𝒪(E[1/S]), with S the size of the sample—which is a random variable for MGH and α-MGH (and also for FracMinHash). Also, the variance for all the different similarity measures mentioned above is 𝒪(E[1/S]).

### Theorem 3.

*Let* $\sigma_{u}$ *be any of the following similarity measures: Jaccard, containment, Kulczinsky 2, Sørensen-Dice, or correlation coefficient. Then*

$$E\left[\sigma_{u}^{\prime }(MGH(A),MGH(B))\right]=\sigma_{u}(A,B),$$

*where* $\sigma_{u}^{\prime }$ *is defined as* σ *with a final “filtering” step. For example, for Sørensen-Dice we have* $SD(A,B)=\frac{2|A\cap B|}{\left|A|+|B\right|}$ *and* $SD^{\prime }(A,B)=\frac{2|MGH(A\cap B)|}{\left|MGH(A)|+|MGH(B)\right|}$. *The filtered versions of the other similarity measures are defined analogously.*

*Let* $\sigma_{b}$ *denote the cosine similarity, or Kulczinsky 1. Then*

$$bias(MGH(A),MGH(B))=E\left[\sigma_{b}^{\prime }(MGH(A),MGH(B))\right]-\sigma_{b}(A,B)=𝒪\left(E\left[\frac{1}{S}\right]\right),$$

*with* S=|MGH(MGH(A)∪MGH(B))|.

*Finally, for any similarity measure* σ *among those mentioned above*

$$V\left[\sigma^{\prime }(MGH(A),MGH(B))\right]=\Theta \left(E\left[\frac{1}{S}\right]\right),$$

*where* S *is as above*.

Hence the bias, if any, the variance, and the mean square error (MSE=bias^2^+variance) of all our estimates based on MGH or α-MGH samples will all go to 0 because E[1/S]=1/E[S]+o(1/E[S]) and E[S]→∞ if n→∞. Details of the proof that E[1/S]∼1/E[S] can be found in Appendix D. In general, it is not true, but because E[S]→∞, plus V[S]=𝒪(E[S]), it is not difficult to obtain such remarkable result.

Irrespective of how we produce the random samples to be used in similarity estimation, the cost of computing such estimates will be linear in the sizes of the samples (plus the time needed to sort the samples, but this is only paid once, or can be omitted if we use hash-join to compute intersections and unions). Therefore, when we have smaller sketches (like in bottom-k) the computation of similarity estimates is extremely fast, but can be quite inaccurate. On the contrary, when the sketches are very large (like in FracMinHash) the estimates are very accurate, but the computation of the estimate is very time-consuming (see, for example, Table 1 in Section 6.4). MGH and α-MGH offer appealing compromises of good accuracy and moderate computational resources.

## 6 Experiments and Results

In this section, we present: 1) simulation experiments verifying theoretical expectations and asymptotically unbiased estimators; 2) a real data experiment highlighting the balance provided by our algorithms.

### 6.1 MaxGeomHash samples grow sub-linearly with the original set

**Fig. 1:**
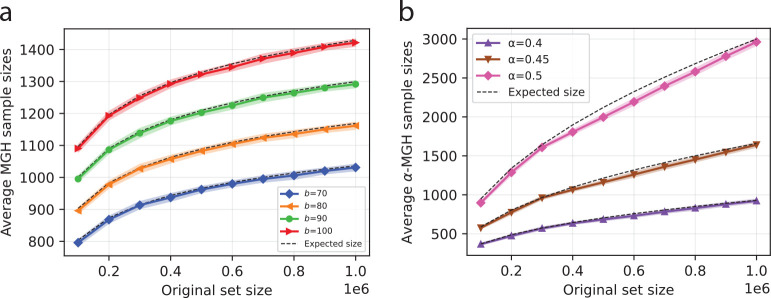
Average sample sizes, recorded from 50 independent trials. Samples are computed using MGH (averages shown in (a)) and α-MGH (averages shown in (b)). The solid lines show the averages, and the shaded regions around the solid lines (very narrow) highlight one standard deviation. Theoretical expectations (excluding lower-order terms) are shown in dashed black lines, closely tracked by the average recorded from simulations.

First, we validate the theoretical expectations of MGH and α-MGH sample sizes using simulated experiments. In these experiments, we generated random sets of 10K to 1M elements consisting of randomly generated alphanumeric strings of length 10. We generated MGH samples with parameters b∈{70,80,90,100}, and α-MGH samples with parameters α∈{0.4,0.45,0.5}. For each choice of b and α, we ran 50 independent trials using different hash seeds and recorded the resulting sample size. The average sample sizes from these 50 trials are shown in Figure 1. The theoretical expectations outlined in Theorems 1 and 2 are also shown in Figure 1 using dashed black lines. When plotting these expected sizes, we excluded the smaller order terms (specifically, $\epsilon_{n,b}$ for the case of MGH, and 𝒪(logn) for the case of α-MGH) from calculation.

The plots clearly show that the growth of the MGH and α-MGH samples aligns closely with the theoretically derived expectation. As the size of the original set grows, the sample size scales sub-linearly, as we expect them to. Figure 1 also shows one standard deviation as shaded regions around the corresponding solid lines. Notably, the variance we observe for MGH and α-MGH samples is quite small – which highlights the stability of the size of the samples produced by MaxGeomHash.

### 6.2 MaxGeomHash samples can estimate similarity across the entire range

We next present experiments investigating the quality of estimated similarity scores using MGH and α-MGH samples and the steps outlined in Section 5. We show only Jaccard estimation results; experiments with other similarity scores were performed, but omitted for brevity.

**Fig. 2:**
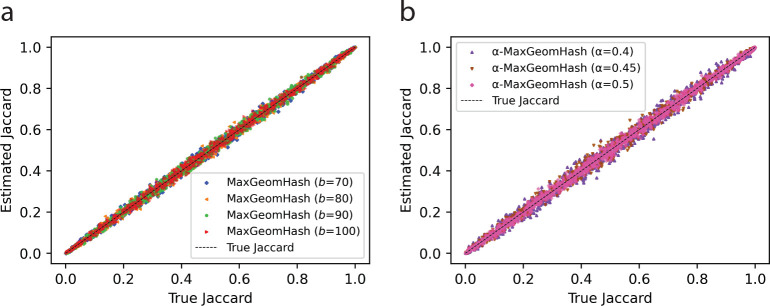
Estimated Jaccard scores against the true Jaccard scores. Estimated Jaccard scores are computed using MGH (shown in Panel a) and α-MGH (shown in Panel b) samples.

We randomly generated 5,000 pairs of sets such that the Jaccard similarity between each pair varied from 0.0 to 1.0. Each individual set contains 100,000 alphanumeric strings of length 10. For each pair, we estimated the Jaccard similarity using MGH samples with parameters b∈{70,80,90,100}, and α-MGH samples with parameters α∈{0.4,0.45,0.5}. In Figure 2, we show these estimated Jaccard values against the true Jaccard scores. Estimated values using MGH samples is shown in Figure 2(a) and α-MGH shown in Figure 2(b). For visual clarity, only a random 1000 points are plotted.

Estimated scores highly correlate with the true values (minimum $R^{2}$ of 0.9932 over all 7 parameter settings). We note slight variability in the estimated Jaccard values, with the variance decreasing as the sketches become larger as b and α become larger. This behavior is expected in sampling/sketching techniques that trade efficiency for tolerable error. Nonetheless, estimated values remain tightly aligned with true scores without systematic bias, indicating that MGH and α-MGH samples provide accurate, unbiased similarity estimation across the entire range.

### 6.3 MaxGeomHash allows asymptotically unbiased similarity estimation while providing a balance between MinHash and FracMinHash

We next compare MaxGeomHash with two other widely used sketching methods, MinHash and FracMinHash. We generated pairs of random sets (A,B) where the number of elements in A and B is the same and the Jaccard similarity between A and B is fixed at 0.5. We varied the number of elements in A and B from 100K to 50M (million). We focus on slightly larger sets (in contrast to the results presented in Figure 1) to capture the range where FracMinHash sketches start to become larger than MinHash sketches.

For each of these (A,B) pairs, we computed sketches using four sketching algorithms: MinHash, FracMinHash, MGH, and α-MGH. For MinHash and FracMinHash, we used the default parameters (k=1000,s=0.001, respectively) set in the software tools Mash [22] and sourmash [9], respectively. These default parameters are widely used in many bioinformatics analyses. To compute MGH and α-MGH sketches, we used b=90, and α=0.45, respectively – which provide a good balance between MinHash and FracMinHash. We note that this specific choice of b=90 and α=0.45 was determined by MinHash and FracMinHash parameters. This parameter choice does not restrict the generality of the results: for any choice of parameters for MinHash and FracMinHash, we can identify b and α values for the two versions of MaxGeomHash that can offer an accuracy-efficiency balance between MinHash and FracMinHash.

**Fig. 3:**
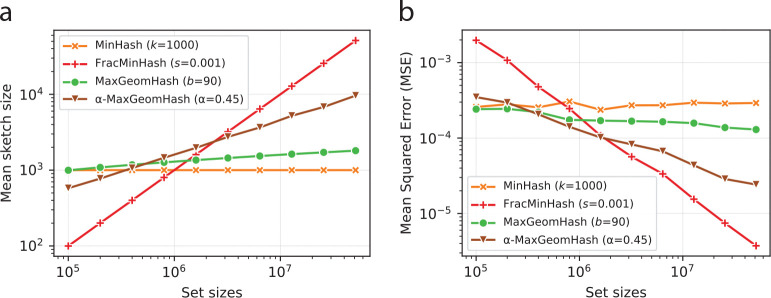
(a) Average sketch sizes for four sketching algorithms against sizes of the original sets, computed from 50 independent trials. (b) Mean squared error (MSE) in estimating the Jaccard similarity of two sets, where the sizes of the two sets are equal. The number of elements in the original sets is shown on the horizontal axis. MSE was averaged from 50 independent hash seeds.

For each algorithm, we ran 500 independent trials. In each trial, we computed sketches of the sets A and B using a distinct seed and estimated the Jaccard similarity using these sketches. The average size of the sketches is shown against the number of elements in A and B in Figure 3a. From the estimated values, we also computed the mean squared error, MSE (note that the true Jaccard is fixed at 0.5), and show how the MSE changes as the sets grow large in Figure 3b.

Panels in Figure 3 show complementary results: as sketch size grows, MSE decreases. MinHash sketches are fixed in size, so MSE remains relatively constant. For the other three algorithms, sketch size grows with |A| and |B|, and MSE diminishes towards zero – indicating that FracMinHash, MGH, and α-MGH are asymptotically unbiased. With the fastest growing sketch size, FracMinHash exhibits the quickest decrease in MSE. MGH and α-MGH sketches grow slower than FracMinHash sketches, so their MSE decays less quickly, while consuming fewer computational resources than FracMinHash.

We repeated this setup for other similarity metrics (cosine, Kulczynski 2) and fixed similarity values (0.1 to 0.9, increments of 0.1), observing similar trends, but omitting for brevity.

### 6.4 An application using real biological data

We conclude with a real biological data application: estimating a similarity tree (a proxy for phylogenetic tree) among ten mammals. The phylogenetic relationships between mammals are well-established [30]. We assess whether k-mer-based sketching techniques can approximate the known phylogeny.

**Fig. 4:**
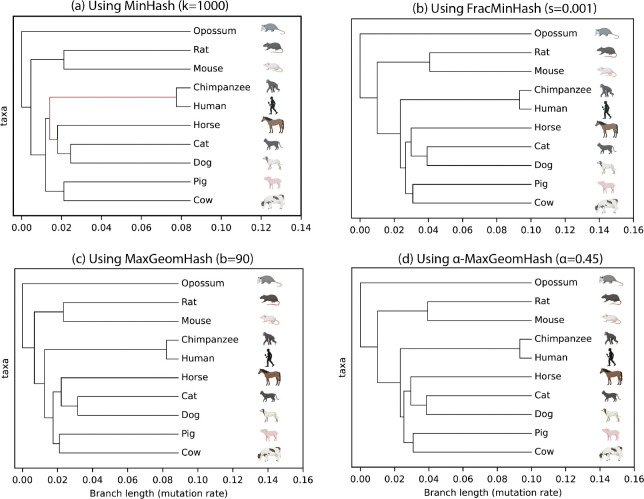
Similarity tree of ten mammal genomes estimated using four sketching methods using k=31-mersketches. The red branch in (a) shows that by using MinHash, Carnivora mammals Cat and Dog are placed close to Primates, whereas in reality, they resemble Pig, Cow, and Horse more, and belong in the Laurasiatheria clade. The other three methods consume more resources and correct this mistake, where MGH and α-MGH offer better resource usage compared to FracMinHash (time & memory listed in Table 1). Icons of the mammals are collected from BioRender.com.

**Fig.5:**
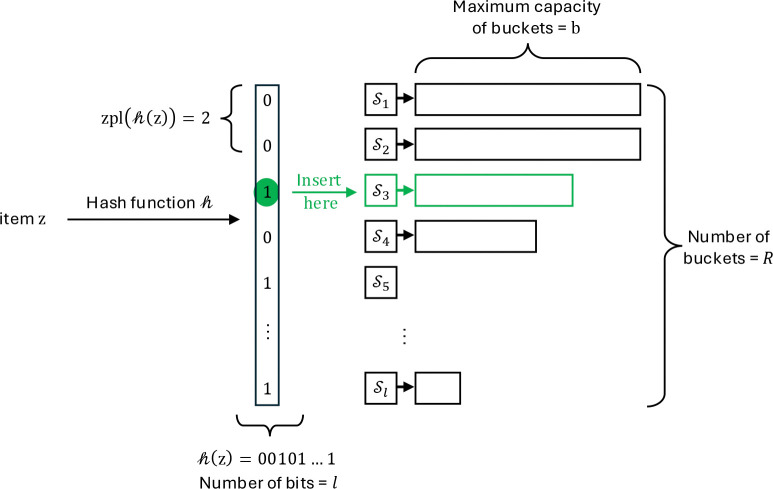
Overview of MaxGeomHash, annotated using the notations defined in Section 3. An l-bit hash function h(.) maps an item z to a bit string of length l. The zero prefix length (zpl) of the hash value h(z) determines which bucket z should be placed in. Each bucket has a maximum capacity of b, which is the integer parameter of MaxGeomHash, where b≥1. Of all items assigned to a bucket, only the items with the b-largest hash values are retained (similar to bottom-k operation, only we are keeping the largest hash valued items, hence the “Max” in MaxGeomHash). The number of non-empty buckets can be at most l, the number of bits in the hash value. At a certain point of execution, only R≤l non-empty buckets exist.

We downloaded ten mammal genome assemblies from Ensembl [32], extracted canonical k-mers (k=31), and computed k-mer-sketches using four algorithms: MinHash, FracMinHash, MaxGeomHash, and α-MaxGeomHash. We used the same parameters as in Figure 3, with motivation detailed in Section 6.3. We computed pairwise Jaccard similarity by loading all sketches in memory, then converted Jaccard scores to genome-wide average mutation rates (i.e., one minus average nucleotide identity, ANI) assuming a simple mutation model using methods from [11]. We then computed a similarity tree from these pairwise mutation rates using UPGMA (Unweighted Pair Group Method with Arithmetic Mean), a standard distance-based method for constructing rooted phylogenetic trees under a molecular clock assumption [6, 20]. Estimated trees for each sketching method are shown in Figure 4.

All methods correctly identify Opossum as the outgroup (most distantly related to the others) and correctly group similar organisms: Rat and Mouse (Rodentia), Human and Chimpanzee (Primates), Cat and Dog (Carnivores), and Pig and Cow (Cetartiodactyla). However, all methods incorrectly place rodents and Primates far apart, though these clades are sister groups.

The major discrepancy between the MinHash tree and the others (Figure 4a) is that with only 1000 hashes, Carnivora (Cat and Dog) are placed close to Primates (Human and Chimpanzee), whereas they actually belong in Laurasiatheria with Pig, Cow, and Horse. Using larger sketches, FracMinHash, MGH, and α-MGH correct this error and are thus more accurate than MinHash. However, FracMinHash requires more computation due to linear sketch growth, whereas MGH and α-MGH reduce computational costs by producing smaller sketches that grow sub-linearly.

Computational resources are reported in Table 1. For fair comparison, we wrote a simple C++ program to read sequences from genome files, split them into k-mers, compute sketches, and store them as plain text files of hash values. FracMinHash is the fastest to compute despite a larger size, since its logic is simple – whether to keep a hash value doesn’t depend on other hash values. The other three methods require maintaining a min (max) heap-like data structure, resulting in slightly higher CPU time. Memory usage for computing sketches is similar across methods, with FracMinHash consuming slightly more due to larger sketches. Disk space for storing sketches starkly highlights size differences: MinHash requires the least space, FracMinHash the most. Finally, computational resources for loading sketches and computing pairwise Jaccard values (and genome-wide average mutation rates) follow sketch sizes, with MinHash consuming the least resources, FracMinHash the most, and MGH and α-MGH providing balance.

We conclude by highlighting that MGH and α-MGH, using parameters b=90 and α=0.45, respectively, can generate clusters that are as accurate as FracMinHash, yet they consume many times less resources when performing pairwise similarity estimation (516x and 22x faster, respectively; 167x and 22x lightweight in memory, respectively; 419x and 22x less storage, respectively). Overall, MaxGeomHash sketches can estimate a similarity tree more efficiently than FracMinHash, and more accurately than MinHash when the parameters are selected carefully. We note that while we used similarity (phylogenetic) tree estimation as an example to highlight this advantage of MaxGeomHash, such benefits can be had in any analysis where sketches are used to estimate similarity/distance.

## 7 Conclusion

We introduced MaxGeomHash and α-MaxGeomHash, both sketching algorithms are one-pass, dependable, order-independent, and mergeable. Without knowing the number of distinct elements n, MGH yields an expected distinct element sample size of Θ(blog(n/b)) while α-MGH yields $\Theta \left(n^{\alpha }\right)$. This situates MGH and α-MGH directly between MinHash (constant sketch size) and FracMinHash (linear sketch size), obtaining the more accurate scaling feature of FracMinHash with the advantage of smaller sketch sizes. We analyzed sample-size expectation/variance and obtained (asymptotically) unbiased estimators for the Jaccard index, and leveraged recent theory results that automatically extend these estimators to a variety of other metrics (containment, cosine, Kulczinsky 1, etc.) as well as provide explicit error bounds and concentration inequalities. These properties make MGH and α-MGH well-suited for the diverse sketching applications that form a core substrate for sequence search, clustering, quality assessment, phylogenetics, and metagenomic surveillance. Workflows that currently use FracMinHash or MinHash (such as Mash screen [21], sourmash gather [12], Skani [27], YACHT [15], fmh-funcprofiler [10], sylph [28], etc.) could be retooled to use MGH to shrink memory/IO budgets without sacrificing accuracy guarantees.

Similarly, recent work relating sketching based metrics to biologically informative metrics like ANI [11], AAI [23], and dN/dS [24] translate directly to MGH: one merely replaces a FracMinHash estimate of these metrics with the MGH counterpart. Recent large-scale computational biology projects, like the Logan effort [2] prioritize storage efficiency. Utilizing MGH in such infrastructures and projects can substantially reduce persistent index size with only modest (and exactly quantifiable) accuracy changes, while retaining compatibility with downstream operations on such sketches.

Future improvements include refining our C++ implementation which is not yet fully optimized. There remains headroom from cache-aware bucket layouts, succinct per-bucket priority structures, SIMD-friendly hash partitioning, and task-parallel refinement. These engineering improvements should further reduce runtime and memory while preserving the theoretical guarantees established for MGH and α-MGH.

## Figures and Tables

**Table 1:** Computational resources used by four sketching algorithms in computing the similarity tree of ten mammals. The trees are shown in Figure 4.

	Computing sketches		Storing sketches	Computing all-pair Jaccard	
Algorithm name	CPU Time (s)	Peak Memory Usage (GB)	Disk space	CPU Time (s)	Memory (MB)
bottom-k MinHash (k=1000)	4673.3	1.397	160 KB	0.01	4.203
FracMinHash (s=0.001)	3970.84	1.421	360 MB	20.64	977.621
MaxGeomHash (b=90)	4365.62	1.397	878 KB	0.04	5.836
α-MaxGeomHash (α=0.45)	4465.38	1.399	18 MB	0.94	43.953

## Footnotes

5 Both MinHash and bottom-k render samples of fixed size, say M. MinHash actually returns M hash values, not the elements themselves: it applies $h_{1},\ldots ,h_{M}$ hash functions to each item in the data set, and keeps the minimum values observed for each of the M hash functions. In bottom-M, we apply a hash function h to each item x in the data set, and we keep the M smallest observed hash values. Despite the two algorithms are different (specially in terms of practical efficiency) they render equivalent random samples of size M, and we will loosely use the terms MinHash and bottom-k interchangeably.

7 It holds for any variant of the algorithm, whether the capacity of the buckets is $b,\left\lceil 2^{\beta i}\right\rceil$ or any function $f_{i}$.

## B Proof of Theorem 1

Recall that for each item $x_{j}$ we compute the position of the leftmost 1 in $h\left(x_{j}\right)$. These follow a geometric distribution $\rho_{j}∼Geom(1/2)$ with $p_{i}:=P\left\{\rho_{j}=i\right\}=2^{-i}$. Denote $X_{i}:=\#\left\{j:\rho_{j}=i\right\}$, the number of items that would be eventually kept in bucket i. Marginally, each $X_{i}∼Binomial\left(n,2^{-i}\right)$ with mean $\mu_{i}=n2^{-i}$, and the vector $\left(X_{1},X_{2},\ldots \right)$ is multinomial $Mult\left(n,{\left(p_{i}\right)}_{i\geq 1}\right)$. But we only keep at most b items on each bucket—those with the b largest hash values—and thus the final size of bucket i is given by $S_{i}=min\left(b,X_{i}\right)$, and that of the returned sample is $S=\sum_{i\geq 1}\;S_{i}$.

### B.1 Expected sample size in MGH

Let i*=lg(n/b). If i* is not integer we may replace it by $\left\lfloor i*\right\rfloor$ (or $\left\lceil i*\right\rceil$) without affecting the nature of the bounds.

We aim to make precise the heuristic argument that we gave in the main body of the paper: if i≤i* then typically $\mu_{i}≳b$, and therefore $S_{i}=b$ with high probability; if i>i* then typically $\mu_{i}\ll b$ and therefore $S_{i}=X_{i}$ with high probability. From that heuristic one expects (crudely)

$$E[S]\approx \sum_{i\leq i*}\;b+\sum_{i>i*}\;\mu_{i}=b\left\lfloor i*\right\rfloor +\sum_{i>i*}\;n2^{-i}=b\left\lfloor i*\right\rfloor +b.$$

Observe the exact identity

$$E\left[S_{i}\right]=b-E\left[{\left(b-X_{i}\right)}_{+}\right]=\mu_{i}-E\left[{\left(X_{i}-b\right)}_{+}\right],$$

where $\left(u{)}_{+}:=max(u,0\right)$. Hence

$$E[S]=\sum_{i\geq 1}\;E\left[S_{i}\right]=\sum_{i\leq i*}\;\left(b-E\left[{\left(b-X_{i}\right)}_{+}\right]\right)+\sum_{i>i*}\;\left(\mu_{i}-E\left[{\left(X_{i}-b\right)}_{+}\right]\right).$$

Define the approximation

$$A:=\sum_{i\leq i*}\;b+\sum_{i>i*}\;\mu_{i}=b\left\lfloor i*\right\rfloor +\sum_{i>i*}\;n2^{-i}=b\left\lfloor i*\right\rfloor +b.$$

The exact error is then

$$err:=E[S]-A=-\sum_{i\leq i*}\;E\left[{\left(b-X_{i}\right)}_{+}\right]-\sum_{i>i*}\;E\left[{\left(X_{i}-b\right)}_{+}\right].$$

Now, for indices below the cutoff i<i*, we can use standard Chernoff bounds to show

$$E\left[{\left(b-X_{i}\right)}_{+}\right]\leq bP\left\{X_{i}<b\right\}\leq b\exp\left(-\frac{{\left(\mu_{i}\;-\;b\right)}^{2}}{2\mu_{i}}\right),$$

and summing over all i<i* gives a negligible contribution $\epsilon_{n}\to 0$. When we are above the cutoff i>i* we have $\mu_{i}<b$ by definition, and we can use the upper-tail bound:

$$E\left[{\left(X_{i}-b\right)}_{+}\right]\leq \mu_{i}P\left\{X_{i}\geq b\right\}\leq \mu_{i}{\left(\frac{e\mu_{i}}{b}\right)}^{b},$$

which also goes to 0 as n grows; thus summing over i>i* gives another negligible contribution. We have therefore only left the critical index i=i*, when $\mu_{i*}=b$. Because we assume b is constant, for n large enough the binomial $X_{i*}$ converges to a Poisson distribution

$$X_{i*}∼Binomial(n,b/n)\underset{n\to \infty \;}{\overset{d\;}{\to }}Poisson(b).$$

and thus

$$E\left[{\left(b-X_{i*}\right)}_{+}\right]⟶E_{b}:=\sum_{m=0}^{b-1}\;(b-m)e^{-b}\frac{b^{m}}{m!}.$$

Combining all three contributions:

$$|err|\leq E_{b}+\epsilon_{n},$$

where $\epsilon_{n}\to 0$ as n→∞, and the statement of the theorem follows.

### B.2 Variance

For the variance, the $S_{i}$’s are not independent but are negatively associated. We can therefore approximate $V[S]≲\sum_{i\geq 1}\;V\left[S_{i}\right]$: the sum of covariances between different indices is negligible because, for large n,b≪n and $X_{i},\;X_{j}$ are almost independent in the Poisson approximation. The contribution of indices i<i* is negligible because $S_{i}=b$ almost surely. Likewise, the contribution of indices i>i* is dominated by the sum of small independent Poisson-like variables, giving roughly b. The errors made by these approximations are collected into some constant $\nu_{b}$, and an upper bound $b^{2}exp(-b/8)$ for its value is not too difficult to establish. Finally, the region around i=i* contributes a finite constant $V_{b}=V[min(b,Poisson(b)]$. This gives a final result in which we haven’t taken into account the slight differences between i* and $\left\lfloor i*\right\rfloor$. When these are taken into account the variance can be shown to be upper bounded by a constant related to b, and we have V[S]=Θ(1).

## C Proof of Theorem 2

### C.1 Expected sample size in α-MGH

We shall use here the same strategy as in the proof of Theorem 1 given in full detail in Appendix B namely, fix the critical value $i*=(1-\alpha )lg\;n=\frac{1}{1+\beta }lg\;n$, where $2^{\beta i*}=\mu_{i*}=\frac{n}{2^{i*}}$, and study the regions where i≪i* and $S_{i}=f_{i}=\left\lceil 2^{\beta i}\right\rceil$ with high probability, where i≫i* and $S_{i}=\mu_{i}<f_{i}$ with high probability, and the “central region” near the critical i*. We will hence have the rough approximation

(1)
$$E[S]\approx \sum_{i\leq i*}\;2^{\beta i}+\sum_{i>i*}\;\mu_{i},$$

which gives us

$$E[S]\approx \frac{2^{\beta \left(i*+1\right)}\;-\;2^{\beta }}{2^{\beta }\;-\;1}+\frac{n}{2^{i*}}=\frac{2^{\beta +1}\;-\;1}{2^{\beta }\;-\;1}n^{\alpha }=\frac{2^{1/(1-\alpha )}\;-\;1}{2^{\alpha /(1\;-\;\alpha )}-1}n^{\alpha },$$

where we use the definition α=β/(1+β). The other approximation given

$$E[S]\approx \left(\frac{1\;-\;\alpha }{\alpha \;ln\;2}+\frac{3}{2}+\frac{\alpha \;ln\;2}{12(1-\alpha )}\right)\cdot n^{\alpha }=:A,$$

comes from using Euler-Maclaurin’s summation formula to evaluate (1), using only the first term of the Bernoulli correction, namely,

$$\sum_{1\leq i\leq i*}\;2^{\beta i}=\int_{1}^{i*}\;2^{\beta x}dx+\frac{2^{\beta i*}\;+\;2^{\beta }}{2}+\frac{B_{2}}{2!}\left(g^{\prime }\left(i*\right)-g^{\prime }(1)\right)+R_{2},$$

with $B_{2}=1/6$ (Bernoulli number), and $g(x)=2^{\beta x}$. We get

$$\begin{array}{c} \int_{1}^{i*}\;\;2^{\beta x}dx\;=\frac{1}{\beta \;ln\;2}\left(n^{\alpha }-2^{\beta }\right)\\ \frac{2^{\beta i*}\;+\;2^{\beta }}{2}\;=\frac{1}{2}n^{\alpha }+2^{\beta -1}\\ \frac{B_{2}}{2}\left(g^{\prime }\left(i*\right)-g^{\prime }(1)\right)\;=\frac{\beta \;ln\;2}{12}\left(n^{\alpha }-2^{\beta }\right), \end{array}$$

and the stated approximation follows. We can explicitly bound the remainder

$$\left|R_{p}\right|\leq {\left(\frac{\alpha \;ln\;2}{2(1\;-\;\alpha )}\right)}^{2p-1}\frac{n^{\alpha }}{\pi^{2p}}=:\epsilon \cdot n^{\alpha },$$

hence we can make ϵ as small as we wish by taking additional terms in the Euler-Maclaurin correction. If we take just one term, as we have done here, $\left|R_{2}\right|\leq 0.035115\ldots n^{\alpha }$.

What remains is to show that err :=E[S]-A=𝒪(logn). First we will do the computations using $2^{\beta i}$ instead of $f_{i}$, and later introduce the corrections needed to cope with $f_{i}=\left\lceil 2^{\beta i}\right\rceil$.

For indices $i\leq \left\lfloor i*\right\rfloor$, we can use Chernoff bounds to show that

$$P\left\{X_{i}\leq 2^{\beta i}-1\right\}\leq exp\left(-c\mu_{i}\right)\leq exp\left(-c2^{\beta i}\right),$$

for some c>0, since $\mu_{i}\geq 2^{\beta i}$ when $i\leq \left\lfloor i*\right\rfloor$, and thus

$$E\left[{\left(2^{\beta i}-X_{i}\right)}_{+}\right]\leq 2^{\beta i}exp\left(-c2^{\beta i}\right),$$

and summing all contributions we get that the contribution to the error coming from $i\leq \left\lfloor i*\right\rfloor$ is 𝒪(1); in particular

$$\sum_{i\geq 1}\;2^{\beta i}e^{-c2^{\beta i}}=𝒪(1).$$

For indices $i>\left\lfloor i*\right\rfloor$, when we are close enough to i* we can use Poisson approximation to get

$$E\left[{\left(X_{i}-2^{\beta i}\right)}_{+}\right]\leq \sum_{t\geq 1}\;P\left\{Poisson\left(\mu_{i}\right)\geq 2^{\beta i}+t\right\}<C_{i}<\infty$$

for some constant $C_{i}$; we need to sum a constant number of such terms, and therefore we will get that their contribution is 𝒪(1). Last but not least, we will consider the region when i>i*+K for some constant K large enough. There we can use the upper tail bound

$$P\left\{X_{i}\geq t\right\}\leq {\left(\frac{e\;\mu_{i}}{t}\right)}^{t},$$

to show

$$E[{\left(X_{i}-2^{\beta i}\right)}_{+}]=\sum_{t=2^{\beta i}+1}^{\infty }\;Pr\left(X_{i}\geq t\right)\leq \sum_{t=2^{\beta i}+1}^{\infty }\;{\left(\frac{e\;\mu_{i}}{t}\right)}^{t}.$$

If we take K large enough, then for i=i*+K we will have

$$q_{i}=\frac{e\mu_{i}}{2^{\beta i}\;+\;1}\leq \frac{1}{4},$$

and thus

$$E[{\left(X_{i}-2^{\beta i}\right)}_{+}]=\sum_{t=2^{\beta i}+1}^{\infty }\;{\left(\frac{e\;\mu_{i}}{t}\right)}^{t}={\left(\frac{e\mu_{i}}{2^{\beta i}\;+\;1}\right)}^{2^{\beta i}+1}\cdot \frac{1}{1\;-\;q_{i}}\leq \frac{4}{3}{\left(\frac{e\mu_{i}}{2^{\beta i}\;+\;1}\right)}^{2^{\beta i}+1}.$$

The bound in the right-hand side decays super-exponentially and hence, the sum of the errors for indices i≫i* is 𝒪(1) too.

But the capacity of the buckets is actually $f_{i}=\left\lceil 2^{\beta i}\right\rceil$ and not $2^{\beta i}$. Since $0\leq f_{i}-2^{\beta i}<1$ for all i, the error that we make using $\sum_{i\leq i*}\;2^{\beta i}$ instead of $\sum_{i\leq i*}\;f_{i}$ is $𝒪\left(i*\right)=𝒪(log\;n)$. For the rest of the argument, we might have used $f_{i}$ everywhere instead of $2^{\beta i}$, the computations would have been messier, but there would have been no essential changes, the error $E[S]-A^{\prime }$ if we used the corrected approximation $A^{\prime }$ with $f_{i}$ (and not the approximation A using $2^{\beta i}$) is still 𝒪(1).

### C.2 Variance

Regarding the variance of S, we can reason as in the analysis of MGH, bounding the total variance by the sum of variances, despite the fact that the $S_{i}$’s are not independent. In the region where i≤i*, we have $S_{i}=f_{i}$ with high probability and $V\left[S_{i}\right]\approx 0$ (for indices i tending towards the low end, there is an exponentially decreasing probability that $S_{i}<f_{i}$). In the tail, when i>i*, we have $S_{i}=X_{i}$ with high probability and $V\left[S_{i}\right]\approx n/2^{i}$. Collecting all the contributions, we get $V[S]=\Theta \left(n^{\alpha }\right)$. Taking into account the errors made by disregarding covariances, the minuscule but non-null probabilities that $S_{i}=X_{i}$ when i≤i* or $S_{i}=f_{i}$ when i>i*, and the effects of ceilings in the definition of $f_{i}$ and of the critical i* will introduce several corrections in our rough estimate, but its asymptotic order of magnitude remains $\Theta \left(n^{\alpha }\right)$.

## D Analysis of $E\left[1/S_{n}\right]$

The variance of the similarity estimators discussed in Section5 is in all cases $\Theta \left(E\left[1/S_{n}\right]\right)$, where we write $S_{n}$ for the size of the samples, stressing the fact that the random variable depends on n. Moreover, for similarity measures such as cosine or Kulczinsky 1, we have asymptotically unbiased estimation, with the bias of order $\Theta \left(E\left[1/S_{n}\right]\right)$.

It is therefore important to know the behavior of $E\left[1/S_{n}\right]$ as n→∞; we show that for the two variants of MGH in this paper we have

(2)
$$E\left[\frac{1}{S_{n}}\right]=\frac{1}{E\left[S_{n}\right]}+o\left(\frac{1}{E\left[S_{n}\right]}\right),$$

and since $E\left[S_{n}\right]\to \infty$ as n→∞, we conclude that the variance tends to 0 for large n, and likewise the bias, if any, also tends to 0.

To show (2) we will use the following: 1) $S_{n}$ is a positive integer value, $0<S_{n}\leq n$, whenever n>0; 2) $\mu_{n}=E\left[S_{n}\right]$ is a non-decreasing function of n and tends to +∞ when n→∞; 3) $\sigma_{n}^{2}=V\left[S_{n}\right]=𝒪\left(\mu_{n}\right)$ for both variants of MGS: it is $V\left[S_{n}\right]=\Theta (b)$ for standard MGH, and $V\left[S_{n}\right]=\Theta \left(n^{\alpha }\right)$ for α-MGH.

Applying Chevbyshev’s inequality, we immediately get that $S_{n}$ has concentration around its mean—we write $S_{n}=\mu_{n}+{𝒪}_{P}\left(\sigma_{n}\right)$, that is, for any ϵ>0, there exists δ:=δ(ϵ) such that

$$P\left\{\left|S_{n}-\mu_{n}\right|\geq \delta \cdot \sqrt{V\left[S_{n}\right]}\right\}\leq \frac{V\left[S_{n}\right]}{\delta^{2}\cdot {\left(\sqrt{V\left[S_{n}\right]}\right)}^{2}}=\frac{1}{\delta^{2}}=\epsilon ,$$

using $\delta =1/\sqrt{\epsilon }$. Because of (2) and (3) above, we have that $\sqrt{V\left[S_{n}\right]}/E\left[S_{n}\right]\to 0$ as n→∞ and $S_{n}=\mu_{n}+{𝒪}_{P}\left(\sigma_{n}\right)$ are equivalent.

Define now $X_{n}:=S_{n}-\mu_{n}$, so that $E\left[X_{n}\right]=0$ and $V\left[X_{n}\right]=E\left[X_{n}^{2}\right]=V\left[S_{n}\right]=\sigma_{n}^{2}$. Note that $-n\leq X_{n}\leq n$ for all n>0. Using a Taylor expansion of $1/S_{n}$ around $\mu_{n}$:

$$\frac{1}{S_{n}}=\frac{1}{\mu_{n}\;+\;X_{n}}=\frac{1}{\mu_{n}}\left(1-\frac{X_{n}}{\mu_{n}}+\frac{X_{n}^{2}}{\mu_{n}^{2}}-\frac{X_{n}^{3}}{\mu_{n}^{3}}+\ldots \right),$$

and taking expectations, we obtain

$$E\left[\frac{1}{S_{n}}\right]=\frac{1}{\mu_{n}}+\frac{\sigma_{n}^{2}}{\mu_{n}^{3}}-\frac{E\left[X_{n}^{3}\right]}{\mu_{n}^{4}}+\ldots$$

The second-order term $\sigma_{n}^{2}/\mu_{n}^{3}=𝒪\left(1/\mu_{n}^{2}\right)$ and higher-order terms are negligible, because the concentration $S_{n}=\mu_{n}+{𝒪}_{P}\left(\sigma_{n}\right)$ and $-n\leq X_{n}\leq n$ guarantee $E\left[X_{n}^{k}\right]=𝒪\left(\sigma_{n}^{k}\right)$ for all k≥2.

## E Methods

In this section, we give the implementation details that were not included in the main text for the sake of brevity.

### E.1 Implementation of MaxGeomHash and α-MaxGeomHash

We implemented MaxGeomHash and and α-MaxGeomHash both using Python and using C++. The Python implementation was used for all the simulation experiments (experiments presented in Sections s to 6.3). The C++ implementation was used to compute the k-mer-sketches of the mammal genomes (experiments presented in Section 6.3). In the Python implementation, we used MurMurHash3 function implemented in the Python package “mmh3” [26]. We used 64-bit unsigned hash values for our purposes, which is a common practice in bioinformatics applications [4, 9]. To maintain the items with the b-largest hash values in the non-empty buckets, we used the package “heapq” in Python. For our C++ implementation, we used a public C++ implementation of the same hash function, and also used unsigned 64-bit hash values. We used priority queues, implemented in the standard template library of C++, to maintain the non-empty buckets in our C++ implementation.

### E.2 Obtaining the mammal genomes

We obtained the ten mammal genomes from Ensembl [32] Release-112. A list of these mammals and their Ensembl IDs is as follows.

**Table 2:** Mammalian reference genomes downloaded from Ensembl (release 112) using wget.

Standard name	Scientific name	Ensembl assembly ID

Human	*Homo sapiens*	GRCh38
Chimpanzee	*Pan troglodytes*	Pan_tro_3.0
House mouse	*Mus musculus*	GRCm39
Norway rat	*Rattus norvegicus*	mRatBN7.2
Dog	*Canis lupus familiaris*	ROS_Cfam_1.0
Cat	*Felis catus*	Felis_catus_9.0
Cow	*Bos taurus*	ARS-UCD1.3
Pig	*Sus scrofa*	Sscrofa11.1
Horse	*Equus caballus*	EquCab3.0
Gray short-tailed opossum	*Monodelphis domestica*	ASM229v1

### E.3 Storing k-mer-sketches as plain text files

We store all sketches in a simple, human-readable plain-text format consisting of a metadata header followed by algorithm-specific data records. Each file begins with several comment lines (prefixed by ‘#’) that record the sketch version, algorithm name, k-mer size, hash seed, hash function, and the parameters relevant to the particular sketching algorithm. After the header, each file lists one record per line. For the MaxGeomHash and α-MaxGeomHash sketch, each line contains four comma-separated fields: the bucket index, the primary hash, a secondary hash used by the geometric sampling process, and a frequency count. This representation encodes all records in the sample produced by MaxGeomHash.

The bottom-k and FracMinHash sketches use simpler formats, each storing a single hash value per line. For bottom-k, the file contains the k smallest hash values observed in the sequence, as specified by the ‘params.k’. For FracMinHash, the file lists all hashes that fall below a threshold derived from the sampling scale parameter (‘params.scale’), effectively implementing downsampling by a fixed fraction. This minimalistic and uniform design across algorithms ensures ease of debugging and straightforward integration into similarity estimation.

### E.4 Comparing pairs of k-mer-sketches

When comparing two sketches, we first check if their metadata indicate compatibility – if hash function or seeds differ, or if k-mer sizes differ, or if the parameters of the sketching algorithm employed to compute the sketches differ, then the two sketches are not compatible with each other, and the similarity estimation does not proceed further. If the two sketches are found to be compatible, the algorithm-specific rules are used to compute a similarity metric, such as the cosine similarity or the Jaccard index.

### E.5 Recording running times, memory usages, and storage usages

We used the standard “/usr/bin/time -v” command to record the CPU time usage, as well as the peak memory usage (maximum resident set size). For the storage usage, the bash utility “find” was used to get the total space required to store the sketches in plain text files.
